# Parathyroid carcinoma arising from auto-transplanted parathyroid tissue after Total Parathyroidectomy in chronic kidney disease patient: a case report

**DOI:** 10.1186/s12882-019-1606-5

**Published:** 2019-11-15

**Authors:** Ho-Ryun Won, Jae Yoon Kang, In Ho Lee, Jin-Man Kim, Jae Won Chang, Bon Seok Koo

**Affiliations:** 1 Department of Otolaryngology-Head and Neck Surgery, Chungnam National University College of Medicine, 640 Daesa-Dong, Chung-Gu, Daejeon, 301-721 Republic of Korea; 2 Department of Radiology, Chungnam National University College of Medicine, Daejeon, Republic of Korea; 3 Department of Pathology, Chungnam National University College of Medicine, Daejeon, Republic of Korea

**Keywords:** Parathyroid carcinoma, Chronic kidney disease, Secondary hyperparathyroidism, Parathyroidectomy, Auto-transplantation

## Abstract

**Background:**

Secondary hyperparathyroidism is a common complication in patients with chronic kidney disease that requires vigilant treatment due to its high mortality rate. Pharmacologic therapy is recommended as an initial treatment; if there is no response, a total parathyroidectomy is performed. In some cases, surgery is accompanied by auto-transplantation of parathyroid tissue.

**Case presentation:**

The patient was diagnosed with chronic kidney disease and received a kidney transplant. However, due to rejection of the transplanted kidney, medical nephrectomy was carried out and routine hemodialysis was initiated and observed. At this time, secondary hyperparathyroidism with elevated parathyroid hormone and hyperphosphatemia developed and pharmacologic treatment was applied. However, there was no response to pharmacologic treatment; therefore, total parathyroidectomy with auto-transplantation was performed. Eight years after surgery, a growing mass was observed in the transplantation site, accompanied by an elevation of parathyroid hormone. A complete resection of the mass was performed, and the patient was diagnosed with parathyroid carcinoma. Additional adjuvant radiation therapy was ordered, and the patient is being monitored.

**Conclusions:**

This is a rare but remarkable case of parathyroid carcinoma arising from auto-transplanted parathyroid tissue after total parathyroidectomy in a patient with secondary hyperparathyroidism. We suggest caution should be taken when choosing to auto- transplant parathyroid tissue and that careful postoperative observation should be performed.

## Background

Secondary hyperparathyroidism is a common complication in patients with chronic kidney disease (CKD) [1]. Prompt treatment of secondary hyperparathyroidism is critical because it causes a mineral disturbance in the body and is associated with an increased mortality rate in CKD patients [2]. Pharmacologic therapy is the first-line treatment; parathyroidectomy is only indicated when pharmacologic treatment is not effective or the side effects are severe [1].

Parathyroid carcinoma is a rare malignant tumor of endocrine origin. The most common cause of parathyroid carcinoma is primary hyperparathyroidism [3]. Other causes of this diseases are chronic stimulation due to secondary or tertiary hyperparathyroidism [4]. Differential diagnosis is important because both atypical parathyroid adenoma and parathyromatosis can occur in secondary hyperparathyroidism patients. The most important point in the differentiation of these diseases is the clinical features such as total serum calcium level and histological features after resection. Especially, parathyroid carcinoma has higher total serum calcium level than other diseases, and histologically, the invasion of surrounding tissues and blood vessels is characteristic [5].

We performed a total parathyroidectomy and auto-transplantation of parathyroid tissue in a CKD patient with secondary hyperparathyroidism who did not respond to pharmacological treatment. Eight years after surgery, a solitary mass was palpated at the site of transplantation of the parathyroid tissue, and parathyroid hormone (PTH) readings continuously increased. A complete excision was performed and the patient was diagnosed with parathyroid carcinoma based on the combined clinical and histological findings. To our knowledge, this is the first case of parathyroid carcinoma arising from auto-transplanted parathyroid tissue in a CKD patient with secondary hyperparathyroidism to be reported. We suggest caution be taken when choosing to auto-transplant parathyroid tissue in CKD patients with secondary hyperparathyroidism, and that careful long-term postoperative observation be performed.

## Case presentation

The patient was diagnosed with type 2 diabetes for the first time at the age of 30; however, the patient was not treated. One year later, the patient visited the Department of Nephrology at Chungnam National University Hospital, where the patient was diagnosed with CKD due to uncontrolled diabetes mellitus and underwent continuous ambulatory peritoneal dialysis. Two years later, the patient received a kidney transplant at a hospital in China at the age of 33. After kidney transplantation, prednisolone, mycophenolate, tacrolimus and/or cyclosporin were used to prevent rejection. However, it was failed to maintain normal renal function; a kidney biopsy conducted a year later showed acute tubular necrosis with interstitial nephritis. After inserting a permanent catheter and undergoing hemodialysis, the patient visited the emergency room after a year due to right lower abdominal pain and was diagnosed with chronic rejection of the transplanted kidney. At age 35, the patient underwent medical nephrectomy of the transplanted kidney through embolization of the renal artery and started routine hemodialysis after receiving arteriovenous fistula surgery in the left forearm. Immunosuppressive therapy was maintained for up to a week after medical nephrectomy, and then discontinued.

During follow-up through an outpatient clinic, the patient presented with hyperphosphatemia (5.5–9.2 mg/dL) and lower limits of normal range of total calcium level (8.6–9.0 mg/d) (Normal range of serum total calcium levels in the laboratory of our institution: 8.7–10.5 mg/d). Based on these findings, the patient was diagnosed with secondary hyperparathyroidism. The patient was given a regimen of calcium acetate, paricalcitol, cinacalcet, and sevelamer carbonate. However, the patient’s PTH level increased to 827.6–1481 pg/mL during drug administration. In addition, hyperplastic parathyroid glands were found on the neck via computed tomography (CT) (Additional file 1 Figure S1). Finally, total parathyroidectomy with auto-transplantation was planned and performed at 38 years of age. A total of 4 hyperplastic parathyroid glands were removed (right superior parathyroid gland:1.5 × 0.9 × 0.5 cm in size, left superior parathyroid gland: 1.0 × 1.0 × 0.7 cm in size, right inferior parathyroid gland: 1.5 × 1.0 × 0.5 cm in size, left inferior parathyroid gland: 2.0 × 1.2 × 0.9 cm in size). After confirming the parathyroid glands through frozen biopsy during operation, some parathyroid gland tissues were collected from left inferior parathyroid gland, and transplanted on the left sternocleidomastoid muscle. In the final pathologic report, all four parathyroid glands showed a histological glandular hyperplasia pattern. Postoperatively, the level of PTH remained at an average of 435.61 pg/mL and the level of phosphorus stabilized to an average of 6.08 mg/dL (Additional file 1 Figure S2).

**Fig. 1:**
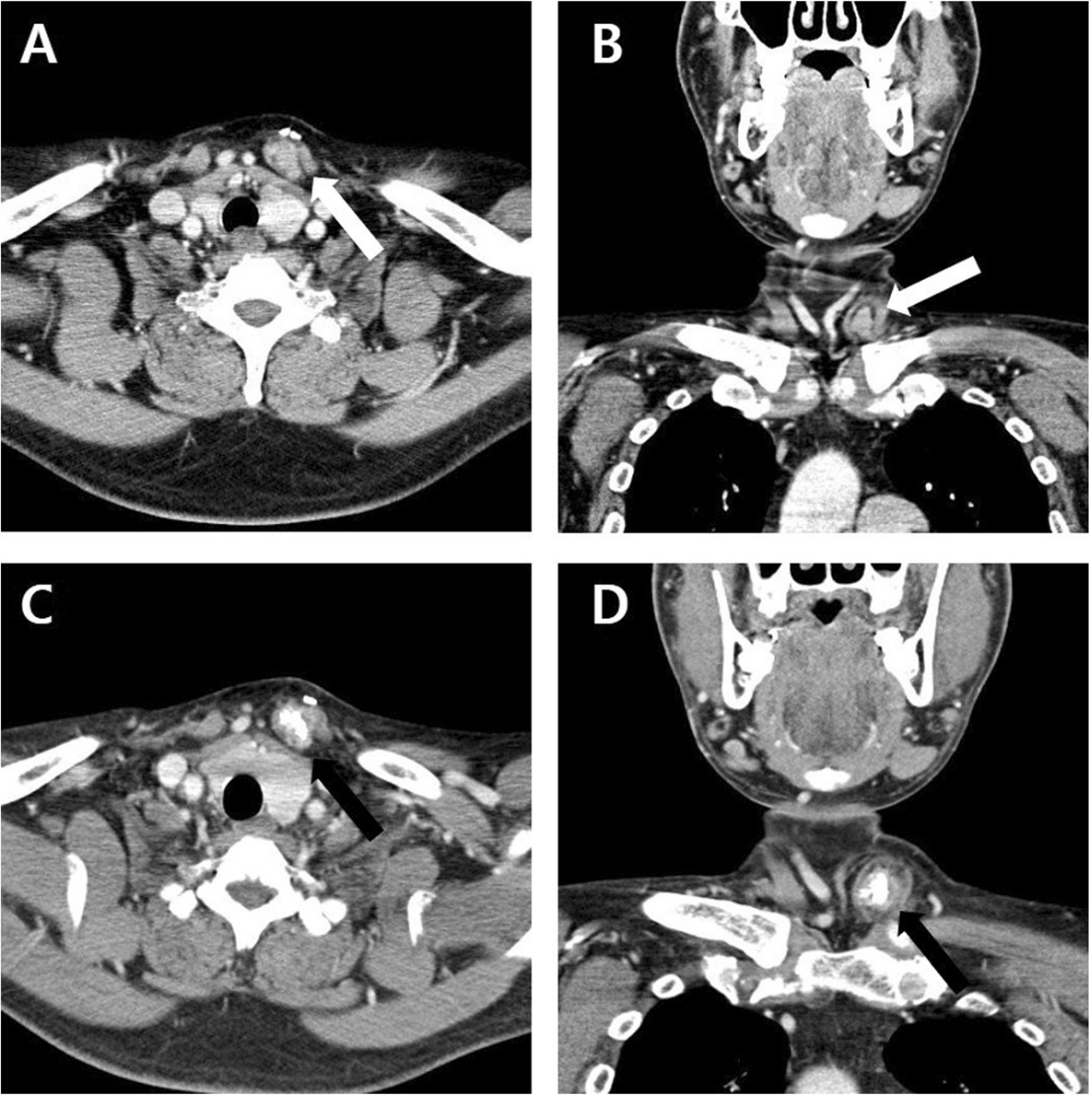
Postoperative follow up neck CT. **a b** An enlarged mass considered as transplanted parathyroid tissue (2.0 × 1.7 × 1.7 cm in size) in the clavicular head of left sternocleidomastoid muscle (white arrow). Enhanced Neck CT performed 3 years after total parathyroidectomy with auto-transplantation. (**a**) Axial view; (**b**) Coronal view; (**c**)(**d**) Compared with 3 years after surgery, a more enlarged mass with internal dense calcification (2.5 × 2.0 × 2.0 cm in size) was found on the clavicular head of left sternocleidomastoid muscle (black arrow). Enhanced Neck CT performed 8 years after total parathyroidectomy with auto-transplantation. (**c**) Axial view; (**d**) Coronal view

**Fig. 2:**
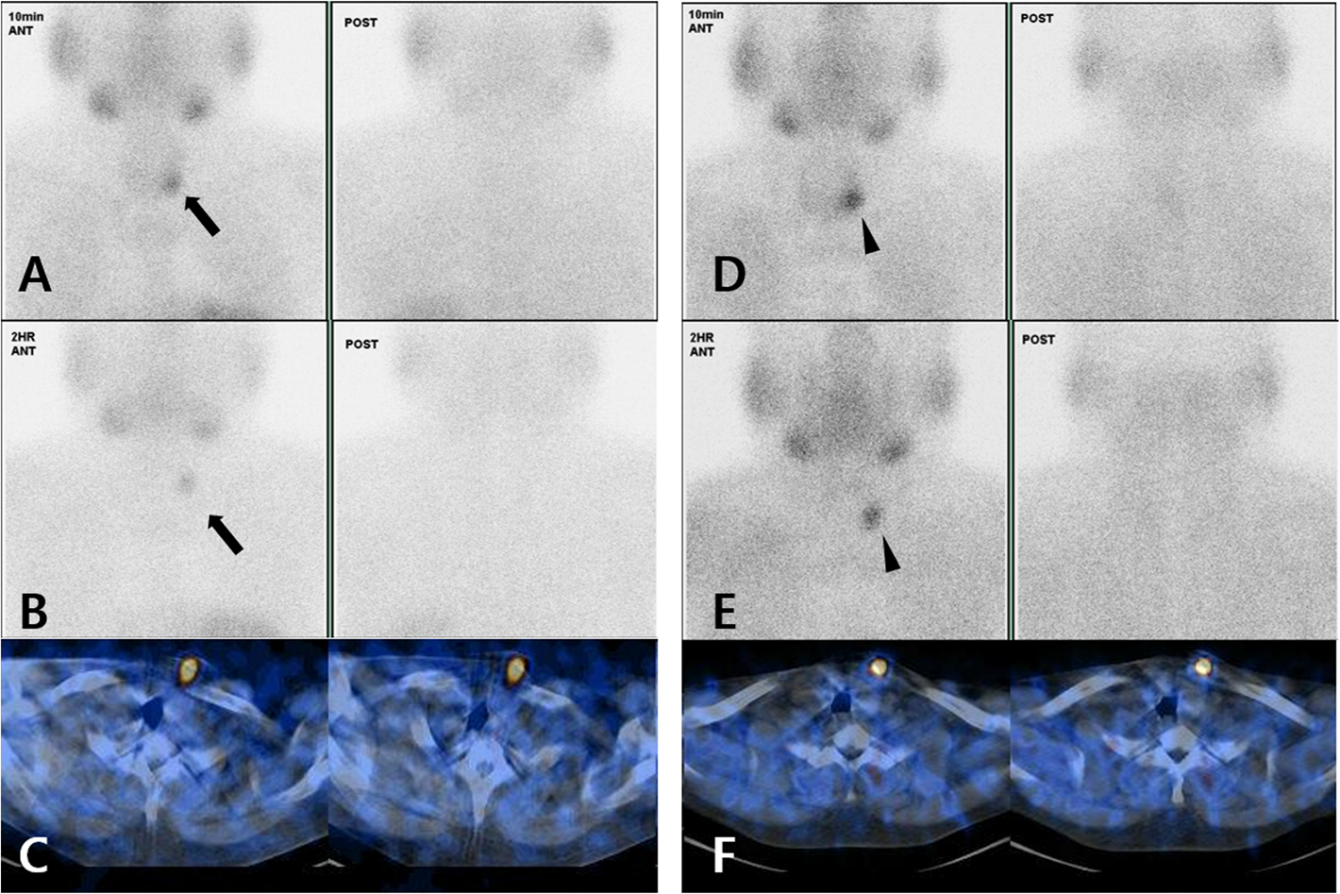
Tc-99 m MIBI dual-phase parathyroid scintigraphy. **a b c**Focal increased uptake in clavicular head area of left sternocleidomastoid muscle (black arrow). Parathyroid Scintigraphy performed 3 years after total parathyroidectomy with auto-transplantation; (A) Early and wash-out images acquired at 10 min post injection; (**b**) Delayed and wash-out images acquired at 120 min post injection; (**c**) Axial fused images acquired at 120 min post injection; (**d**) (**e**) (**f**) A hyperfunctioning auto-transplanted parathyroid tissue with increased uptake was identified compared to previous scintigraphy (black arrowhead). Parathyroid Scintigraphy performed 8 years after total parathyroidectomy with auto-transplantation; (**d**) Early and wash-out images acquired at 10 min post injection; (**e**) Delayed and wash-out images acquired at 120 min post injection; (**f**) Axial fused images acquired at 120 min post injection

However, 8 years after surgery, the patient’s PTH level began fluctuating between 485 and 1399 pg/mL and his total calcium rose to an average of 9.8 mg/dL intermittently. However, normal levels of blood phosphorus were maintained (5.34 mg/dL) (Additional file 1 Figure S2). In addition, a solid, fixed, 3 cm mass was palpated at the implant site. A CT scan of the neck was performed and confirmed that the auto-transplanted parathyroid tissue had become enlarged and densely calcified (Fig. 1c, d). It had also increased to 2.5 × 2.0 × 2.0 cm in size, which was larger than what was seen at the 3-year follow-up exam (Fig. 1a, b). Tc-99 m MIBI dual-phase parathyroid scintigraphy showed that the auto-transplanted parathyroid tissue was hyperfunctioning (Fig. 2d-f) compared to the 3-year follow-up (Fig. 2a-c) [6].

**Fig. 3:**
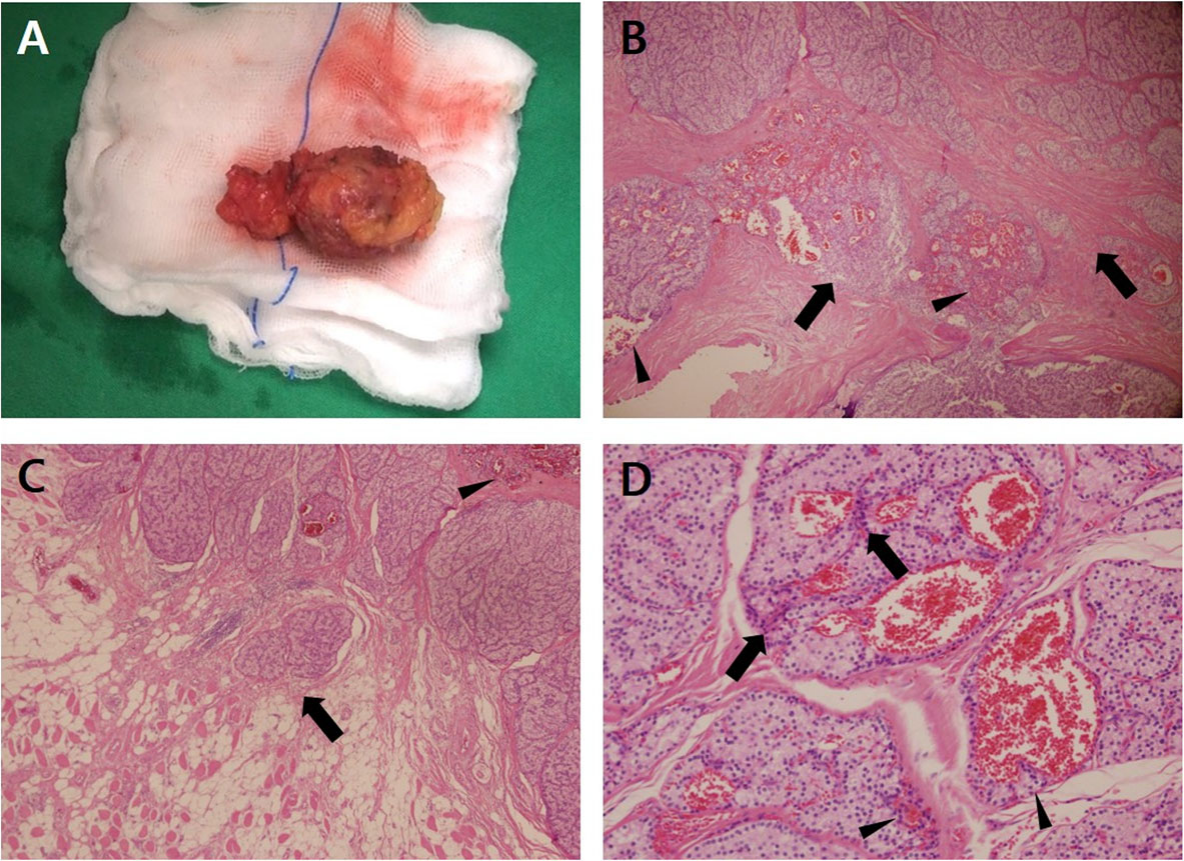
Gross and pathologic findings of extirpated auto-transplanted parathyroid tissue. **a** Gross findings. A mass of about 2 × 2 cm in size with solitary and irregular margins was identified; (B-D) Pathologic findings. **b** Invasion into surrounding muscular structure(black arrow) and vessels (black arrowhead) (H & E, X 100); (**c**) Satellite lobule (black arrow) and partial necrotic finding (black arrowhead) (H & E, X 100); (**d**) Highly mitotic feature of parathyroid cell (black arrow) and minimally invasion into vascular structure (black arrowhead) (H & E, X 200)

A complete resection of the auto-transplanted parathyroid tissue was performed at age 46. The excised mass was 2 × 2 cm in size with irregular margins and a calcareous composition (Fig. 3a). The final pathologic diagnosis was parathyroid carcinoma accompanied by surrounding muscle and vascular invasion (Fig. 3a-d). The resection margin was clear but had a closed margin (safety resection margin, 1 mm). There was no local or distant metastasis on positron emission tomography-CT. Therefore, additional radiotherapy was ordered. Five months after complete resection of the auto-transplanted parathyroid gland, the patient has completed adjuvant radiotherapy without specific complications or symptoms. PTH level remains stable.

## Discussion and conclusions

The incidence of CKD is rising steadily with the increased prevalence of diabetes and hypertension [7]. The severity of CKD is graded according to the glomerular filtration rate (GFR) [8]. Secondary hyperparathyroidism is a common complication in patients with CKD, especially in patients receiving dialysis [9]. Treatment of secondary hyperparathyroidism is important because it plays an important role in mineral bone disease and cardiovascular diseases associated with CKD [9, 10]. Despite many advances in dialysis treatment, there has been no significant change in the mortality rate of CKD patients, and the disturbance in mineral and bone metabolism by secondary hyperparathyroidism is a major cause of such mortality [11–13].

Secondary hyperparathyroidism in CKD patients is caused by hyperphosphatemia, which is a major pathophysiologic mechanism [7]. The reduction in GFR leads to a decrease in phosphorus clearance, which results in phosphorus retention. In this hyperphosphatemic state, 1,25-dihydroxyvitamin D is decreased by the increase in fibroblast growth factor 23 [14–16]. This eventually leads to hypocalcemia, which stimulates the parathyroid to induce continuous secretion of PTH [7, 15, 17]. This is a normal regulatory response in low-grade CKD. However, if the effect of PTH on phosphorus reabsorption is reduced due to the deterioration of renal function, PTH continuously increases and eventually causes a disturbance in mineral and bone metabolism [7, 15]. The increased phosphorus causes further secretion of PTH, and PTH persists in a vicious cycle leading to hyperphosphatemia [7].

Pharmacological intervention is considered the first-line treatment for secondary hyperparathyroidism. Pharmacologic therapy is based on calcimimetics, calcitriol, and vitamin D analogs, including vitamin D mimetics [18]. Phosphorus binders for the control of hyperphosphatemia are also used for treatment [19, 20]. Parathyroidectomy is only considered when there is no response to pharmacologic therapy or when its side effects are too severe [21]. According to recent 2017 Kidney Disease: Improving Global Outcomes guidelines, parathyroidectomy is indicated for patients at all stages of CKD ranging from early GFR decline (60 ml/min per 1.73 m^2^) to dialysis when there is no response to pharmacologic therapy. In addition, the incidence of parathyroidectomy is gradually increasing [22]; one study reported that parathyroidectomy was performed in 15% of patients who received dialysis for 10 years and in 38% of patients who received dialysis for 20 years [23].

Total parathyroidectomy with or without auto-transplantation or subtotal parathyroidectomy are the surgical methods of choice for the treatment of secondary hyperthyroidism. Total parathyroidectomy with auto-transplantation is preferred for patients who have reasons to avoid subsequent surgeries or long-term hemodialysis after surgery [15, 24]. Total parathyroidectomy alone is useful for preventing the recurrence of secondary hyperparathyroidism in patients who do not have the potential for kidney transplantation and who have a long-life expectancy [25]. In this case study, the patient’s secondary hyperparathyroidism persisted despite pharmacologic treatment, and surgical treatment was indicated. Although there was a risk of recurrence, total thyroidectomy with auto-transplantation was performed due to the possibility of future kidney re-transplantation. Parathyroid carcinoma is a rare malignant tumor; the main cause is primary hyperparathyroidism [3]. Parathyroid carcinoma is commonly sporadic and may be associated with familiar primary hyperparathyroidism or jaw tumor syndrome [3, 5]. Rarely, a history of cervical irradiation or chronic stimulation, such as secondary hyperparathyroidism, is known to be the cause of parathyroid carcinoma [26].

Parathyroid carcinoma in patients with secondary hyperparathyroidism has been reported very rarely [4, 27], and parathyroid carcinoma in auto-transplanted parathyroid tissue has not been reported previously. Parathyromatosis is also a rare disease, but it is still more common in patients with secondary hyperparathyroidism than parathyroid carcinoma [28, 29]. Differential diagnosis between parathyroid carcinoma and parathyromatosis is an important focus. In our case, the mitotic features of the parathyroid cells were prominent under light microscopy, and the invasion of muscle and blood vessels was remarkable. Therefore, the pathologic diagnosis was confirmed as parathyroid carcinoma. Of course, these pathologic findings are not the definitive point of differential diagnosis between parathyroid carcinoma and parathyromatosis [5]. In this case, clinically, the patient was not hypercalcemic (> 14 mg/dL) but 10 mg/dL is relatively high for a hypocalcemia-associated CKD patient. In addition, the lesion appeared in the form of a solitary mass rather than multinodular, which is the characteristic form of parathyromatosis in auto-transplanted parathyroid tissue [29]. These findings ultimately confirmed the diagnosis of parathyroid carcinoma, and adjuvant radiation therapy was ordered and performed.

This patient is the first reported case of parathyroid carcinoma arising from auto-transplanted parathyroid tissue after total parathyroidectomy resulting from secondary hyperparathyroidism. Although the disease incidence is rare, it is a result that cannot be overlooked in long-term dialysis patients, in whom chronic stimulation of parathyroid tissue is inevitable. Likewise, parathyromatosis, which occurs rarely in auto-transplanted parathyroid tissue, is considered a low-grade malignancy. Therefore, if total parathyroidectomy with auto-transplantation is performed in patients with secondary hyperparathyroidism, continuous treatment and careful observation of the transplant site is necessary. In conclusion, we provisionally recommend total parathyroidectomy would be performed without auto-transplantation in secondary hyperparathyroidism patients, especially younger patients who have a long-life expectancy or those who are less likely to undergo renal transplantation as well as all patients who do not necessarily require auto-transplantation.

## Supplementary information

**Additional file 1: Figure S1.** Preoperative neck CT. (A) (B) Small enhancing masses in the retrothyroidal area on both sides (black arrows). (A) Axial view; (B) Coronal view. **Figure S2.** Serologic test results. Elevated PTH and phosphorus decreased after total parathyroidectomy with auto-transplantation (1st operation: 10/2011). Prior to complete resection (2nd operation: 03/2019) of the auto-transplanted parathyroid tissue, PTH and total calcium levels were increased while phosphorus remained normal. After complete resection, PTH and calcium levels decreased. All measurements represent the mean value.

## Data Availability

The datasets used and/or analyzed during the current study available from the corresponding author on reasonable request.

## Abbreviations

**CKD**: Chronic kidney disease
**CT**: Computed tomography
**GFR**: Glomerular filtration rate
**PTH**: Parathyroid hormone
