# Supplementary material for: Parathyroid carcinoma arising from auto-transplanted parathyroid tissue after Total Parathyroidectomy in chronic kidney disease patient: a case report
Source: BMC Nephrol. 2019 Nov 15;20:414. doi: 10.1186/s12882-019-1606-5 (PMC6858716; doi:10.1186/s12882-019-1606-5)
Supplement: Supplementary file 1 — Additional file 1: Figure S1. Preoperative neck CT. (A) (B) Small enhancing masses in the retrothyroidal area on both sides (black arrows). (A) Axial view; (B) Coronal view. Figure S2. Serologic test results. Elevated PTH and phosphorus decreased after total parathyroidectomy with auto-transplantation (1st operation: 10/2011). Prior to complete resection (2nd operation: 03/2019) of the auto-transplanted parathyroid tissue, PTH and total calcium levels were increased while phosphorus remained normal. After complete resection, PTH and calcium levels decreased. All measurements represent the mean value. [file 12882_2019_1606_MOESM1_ESM.pdf]

Supplementary Figure 1

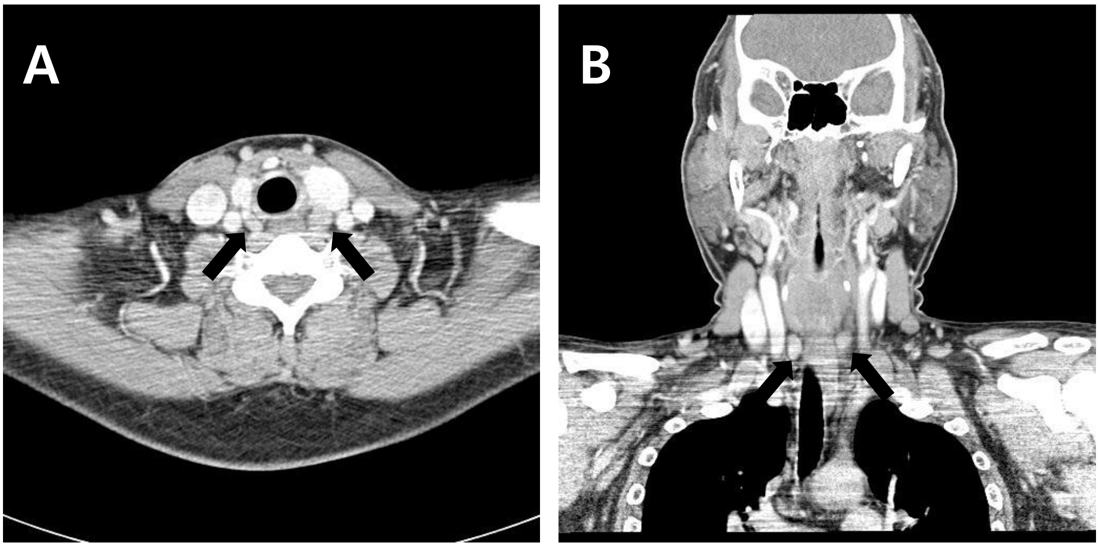

Supplementary Figure 1: Preoperative neck CT. (A) (B) Small enhancing masses in the retrothyroidal area on both sides (black arrows). (A) Axial view; (B) Coronal view.

Supplementary Figure 2

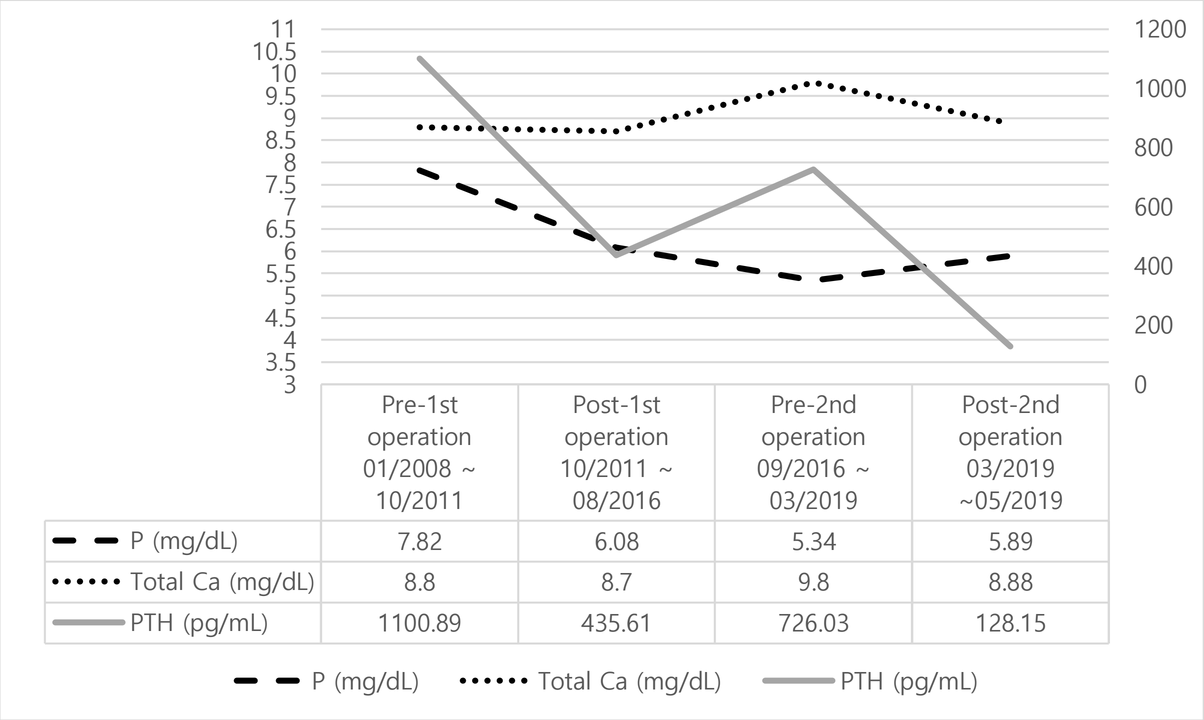

	Pre-1st operation 01/2008 ~ 10/2011	Post-1st operation 10/2011 ~ 08/2016	Pre-2nd operation 09/2016 ~ 03/2019	Post-2nd operation 03/2019 ~05/2019
P (mg/dL)	7.82	6.08	5.34	5.89
Total Ca (mg/dL)	8.8	8.7	9.8	8.88
PTH (pg/mL)	1100.89	435.61	726.03	128.15

Supplementary Figure 2: Serologic test results. Elevated PTH and phosphorus decreased after total parathyroidectomy with auto-transplantation (1st operation: 10/2011). Prior to complete resection (2nd operation: 03/2019) of the auto-transplanted parathyroid tissue, PTH and total calcium levels were increased while phosphorus remained normal. After complete resection, PTH and calcium levels decreased. All measurements represent the mean value.
